# PETALS: Proteomic Evaluation and Topological Analysis of a mutated Locus' Signaling

**DOI:** 10.1186/1471-2105-11-596

**Published:** 2010-12-13

**Authors:** Gurkan Bebek, Vishal Patel, Mark R Chance

**Affiliations:** 1 Center for Proteomics and Bioinformatics, Case Western Reserve University, 10900 Euclid Ave, Cleveland OH, 44106, USA; 2 Case Comprehensive Cancer Center, Case Western Reserve University, 10900 Euclid Ave, Cleveland OH, 44106, USA; 3 Genomic Medicine Institute, Cleveland Clinic, 9500 Euclid Avenue, Cleveland OH, 44195, USA; 4 Department of Genetics, Case Western Reserve University, 10900 Euclid Ave, Cleveland OH, 44106, USA; 5 Department of Physiology and Biophysics, Case Western Reserve University, 10900 Euclid Ave, Cleveland OH, 44106, USA

## Abstract

**Background:**

Colon cancer is driven by mutations in a number of genes, the most notorious of which is *Apc*. Though much of *Apc*'s signaling has been mechanistically identified over the years, it is not always clear which functions or interactions are operative in a particular tumor. This is confounded by the presence of mutations in a number of other putative cancer driver (CAN) genes, which often synergize with mutations in *Apc*.

Computational methods are, thus, required to predict which pathways are likely to be operative when a particular mutation in *Apc *is observed.

**Results:**

We developed a pipeline, PETALS, to predict and test likely signaling pathways connecting *Apc *to other CAN-genes, where the interaction network originating at *Apc *is defined as a "blossom," with each *Apc*-CAN-gene subnetwork referred to as a "petal." Known and predicted protein interactions are used to identify an Apc blossom with 24 petals. Then, using a novel measure of bimodality, the coexpression of each petal is evaluated against proteomic (2 D differential In Gel Electrophoresis, 2D-DIGE) measurements from the *Apc*^*1638N*+/-^mouse to test the network-based hypotheses.

**Conclusions:**

The predicted pathways linking *Apc *and *Hapln1 *exhibited the highest amount of bimodal coexpression with the proteomic targets, prioritizing the *Apc-Hapln1 *petal over other CAN-gene pairs and suggesting that this petal may be involved in regulating the observed proteome-level effects. These results not only demonstrate how functional 'omics data can be employed to test in *silico *predictions of CAN-gene pathways, but also reveal an approach to integrate models of upstream genetic interference with measured, downstream effects.

## Background

It is clear that sporadic colorectal cancer - as well as other cancers - is largely the product of acquired somatic mutations [1]. Though many of these mutations are functionally relevant to the tumor ("driver" genes), the most well-studied cancer driver gene remains *Apc *(adenomatous polyposis coli), thought to be the first hit in the majority of nonhereditary colon cancers [2]. While *Apc *is commonly known as an antagonist to *β*-catenin and WNT signaling, a growing body of evidence points to the importance of *Apc *in a variety of other cellular contexts - from microtubule polymerization [3] to cell migration [4]. *Apc *also plays important roles in chromosome segregation and stability, localizing to spindles, kinetochores, and centrosomes in mitosis [5,6]. The myriad aspects of *Apc *signaling may not be relevant in all cellular contexts, however, as signaling depends upon the background gene expression program and, in cancer biology, is often the result of multiple mutations. In fact, mouse models mutated at two driver genes simultaneously have shown a synergistic (i.e. non-additive) increase in tumor burden, such as in *Pten-Apc *[7], *Kras-Tgfb *[8], and *Apc-Trp53 *[9] double mutants. Such genetic synergy suggests that the pathways emanating from the two genes intersect downstream, supporting the idea that only a subset of all possible pathways are involved in a tumor harboring a mutation in *Apc*. We hypothesize that these mutations have distinct synergistic effects on the cancer phenotype, such that the activities of these networks are greatly associated with the measured downstream changes in the proteome of the intestine. We argue that these measured molecular changes can be leveraged to elucidate which pathways are most relevant to the disease model at hand.

**Figure 1:**
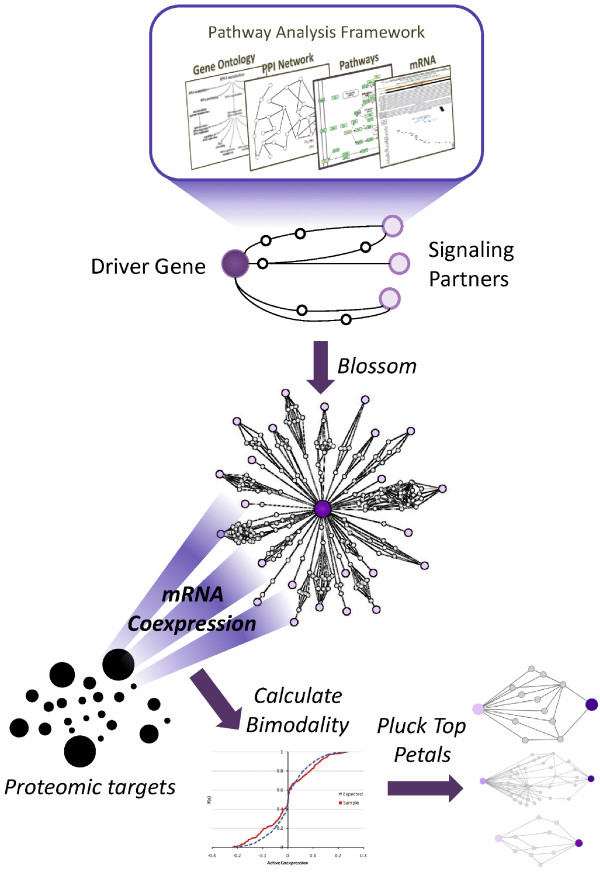
**Workflow for PETALS: Proteomic Evaluation and Topological Analysis of a Mutated Locus' Signaling**. We begin with a cancer driver gene of interest (e.g. *Apc*) and a set of putative signaling partners. The pathways between these two sets are predicted based on protein-protein interactions, coupled with mRNA coexpression and GO annotations. The prediction of pathways to the various signaling partners allows individual subnetworks to blossom into a flower with many *petals*. The biological relevance of each petal is assessed against proteomic evidence (i.e. 2D-DIGE), using the bimodality of mRNA coexpression to quantify this association. This results in a ranking of petals, which can then be plucked for further experimental evaluation.

In order to prioritize the various pathways associated with a cancer driver gene, we have developed a computational framework to first predict the set of pathways functionally related to *Apc *signaling in mouse models (Figure 1). Our algorithm mines chains of proteins (simple paths) from a protein-protein interaction (PPI) network; these paths are then filtered by tissue-specific mRNA coexpression and Gene Ontology (GO) [10] annotation rule mining [11]. To identify biologically relevant paths, we constrain our search space to pathways connected to previously identified cancer driver genes (CAN-genes) [12], as many of these pairings are expected to be simultaneously mutated. The set of paths linking *Apc *to each CAN-gene comprises a subnetwork, which we refer to as a *petal *in the *Apc blossom*. As each petal is based on in silico predictions, we then use publicly available functional genomic and proteomic data from the intestine of the *Apc*^*1638N*+/- ^mouse to assess the biological relevance of each petal in this mouse model. As proteins themselves are the mediators of cellular functions, we mapped proteome-level measurements identified through 2 D differential In Gel Electrophoresis (2D-DIGE) to each petal, using mRNA-level coexpression to quantify the strength of the relationship. We chose to use 2D-DIGE - a widely used 2 D gel electrophoresis based method - to illustrate our approach. However, our methods can utilize a variety of proteomics data (e.g. label-free LC/MS (Liquid Chromatography/Mass Spectrometry), protein antibody chips etc.). Though transcriptional activity (i.e. mRNA level) does not strictly correlate with translational activity (i.e. protein level) [13,14], coexpression information can still be helpful in uncovering regulatory hot spots in protein networks [15]. Testing each petal against such functional data correlates gene and protein expression readouts with specific driver gene relationships, thereby allowing the experimenter to identify the petal most likely to be operative in this particular mouse model.

## Results and Discussion

In this paper, we present a method to capture the likely signaling pathways of a cancer driver gene, focusing on the signaling related to *Apc *as an example. The initial set of pathway predictions are mined from protein-protein interaction networks, coupled to mRNA coexpression data and Gene Ontology association rules. We refer to this data-mining process as the Blossom Algorithm (Figure 1 top), as it produces a network connecting a driver gene (e.g. *Apc*) to a set of putative signaling partners, referred to as the *Apc *blossom (Figure 1 center). The *Apc *blossom is then pruned using biological evidence (microarray and proteomic data) to identify a candidate petal, or subnetwork, most likely to be involved in *Apc *signaling (Figure 1 bottom).

### The Apc Blossom: A PETALS Network

**Figure 2:**
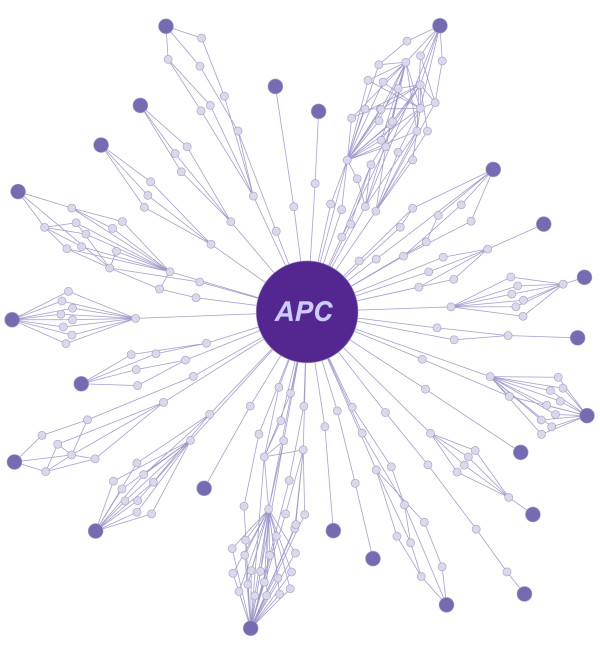
**The Apc blossom**. Using the Blossom algorithm, we search for paths in the filtered and imputed PPI network that connects *Apc *to other CAN-genes [12]. For the CAN-genes that possess at least one path to *Apc*, this resulted in 24 petals (*p *< 0.05) - one petal for each CAN-gene.

To study CAN-gene pathways operative in the *Apc*^*1638N*+/- ^mouse model, we used the Blossom algorithm to identify pathways connecting *Apc *to 68 other CAN-genes [1,12]. In summary, the Blossom Algorithm mines publicly available protein-protein interaction networks to uncover paths - i.e. chains of proteins - likely to be "functional." As evidence of a path's functionality, we use mRNA coexpression and Gene Ontology association rules. As our current knowledge of molecular networks is incomplete [16], we use sequence homology to infer these missing data. The details of the Blossom algorithm follow below (see Methods for additional details; refer to Figure 1 in [17] for a diagram). First, likely false positives from the underlying PPI network are filtered out. Next, using this filtered PPI network, we were able to find paths linking *Apc *to 42 of the CAN-genes, forming subnetworks, which we refer to as *petals*. After imputing interaction edges using sequence homology [11], this number was increased to 65. However, filtering out paths whose (*i*) average mRNA coexpression was low (*r *< |0.6|, a significance threshold validated in similar studies [11,17]) and (*ii*) support of GO annotation association rules based on known signaling pathways and functional annotations [11] was weak (*p *- *value *> 0.05), the number of *Apc*-CAN-gene petals was reduced to 24 (Figure 2). The petals identified vary in the number of nodes (from 3 - 35) and edges (from 2 - 80) they contain, with some nodes beings shared among multiple petals.

A blossom can be constructed for a wide variety of genes, with the stipulation that corresponding microarray data is available. In our case study of *Apc*, we employ mRNA expression data from intestinal tumors harvested from *Apc*^*Min*/+ ^mice. As multiple mutations are present in these samples, coexpression measurements calculated for this dataset are representative of the tumor microenvironment; as such, both *Apc *signaling, as well as additional CAN-gene signaling, are likely to be active simultaneously. While the presence of these multiple, active pathways increases the signal associated with cross-talk within in each petal, it does not allow us to determine which pathways are most strongly associated with *Apc *signaling alone. To answer this question, as outlined in the next section, we used mice with a particular heterozygous mutation in *Apc *- 1638N - that results in a mild intestinal cancer phenotype [18], thereby minimizing the noise arising from the many pathways activated in a full-blown tumor. Since we are interested in assessing the systems-level effects of such mutations, we focus on measuring the downstream effects of these genes via 'omic experiments.

### Plucking Petals: Testing the Bimodality of Coexpression

**Figure 3:**
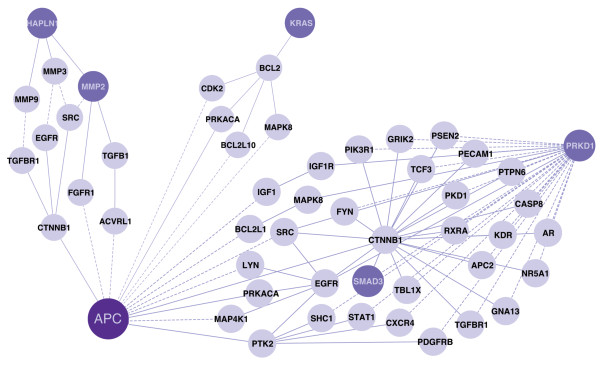
**Top three significant Apc petals**. The top three petals, *Apc-Hapln1 *(left, *p *= 0.0068), *Apc-Kras *(middle, *p *= 0.0157), and *Apc-Prkd1 *(right, *p *= 0.0167), found to be significant after coexpression correlation with proteomic targets are shown. The darker nodes represent CAN-gene proteins. When searching for paths, CAN-genes were not differentiated from other proteins on the network, hence multiple CAN-genes exist in some of the petals. The dashed edges represent novel interactions predicted to exist on the network, whereas solid edges are known interactions.

**Figure 4:**
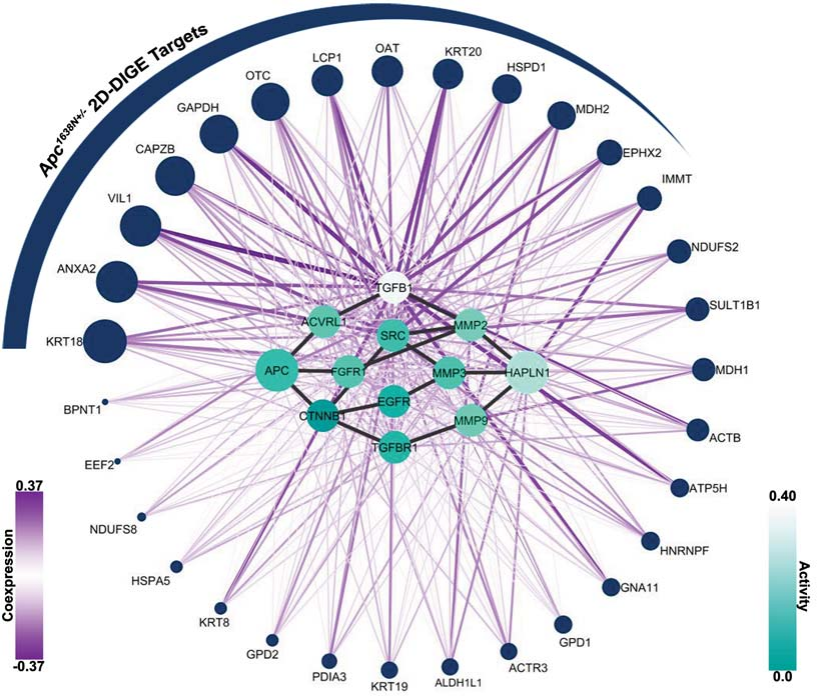
**2D-DIGE Proteomics targets and top scoring Apc-Hapln1 petal**. The *Apc-Hapln1 *petal is illustrated in the center, with the 2D-DIGE targets shown on the periphery. The activity (turquoise) of each node in the petal ranges from 0.0-0.4, and their coexpression links (purple) range from -0.37-0.37. The size of the 2D-DIGE targets corresponds to their degree; for each 2D-DIGE target, this is calculated as the sum of the absolute value of coexpression links.

The *Apc*^*1638N*+/- ^mouse model represents a perturbation of the stamen (the center node) in the *Apc *blossom, and such a perturbation is expected to have far-reaching molecular effects. This was supported by the 2D-DIGE proteomic experiments that identified 31 proteins with a variety of cellular functions from the intestinal epithelium of compared to wild-type. We hypothesized that if one of the petals in the blossom truly reveals signaling associated with this mutation of *Apc*, then the nodes of this petal are more likely to associate with the 2D-DIGE targets than a random group of proteins. To gauge this association, we used a map of coexpression compiled from the corresponding *Apc*^*1638N*+/- ^intestinal epithelium mRNA-expression profile. Assuming that the signaling molecules in a petal are upstream of the 2D-DIGE targets, strong coexpression between a petal and the 2D-DIGE targets can help to identify the causative signaling events that led to these measured changes in abundance of the proteome. Since coexpression is most informative when it relates to differentially expressed nodes (i.e. those that differ between the mutant and wild-type mice), we modulated the coexpression values associated with the nodes in each petal by their respective levels of differential expression. This allows for the identification of nodes where any individual node may have a low level of expression, but the collective level of expression across nodes may be high. We further posited that, if a group of proteins truly is coregulated, then we expect to see deviations in the tails of the coexpression distribution when compared to the expected (background) distribution. To gauge this deviation, we introduced the bimodality, *β*, of coexpression: a measure based on the mass (i.e. area under the curve) of the cumulative distribution functions' and the distance of the mass from the origin. This allowed us to prioritize the petals by their respective *p*-values and the top three petals are shown in Figure 3 (See Additional File 1 Table 1 for the complete list). In Figure 4 the 31 2D-DIGE targets are shown on the periphery of the petal, ranked by their degree (i.e. sum) of absolute coexpression. This representation also facilitates the prioritization of 2D-DIGE targets, placing emphasis on those targets whose regulation is supported by multiple elements of the candidate petal. Much of the coregulation can be explained by a few key signaling intermediates - notably, *TGFB1*, which has both a high level of differential expression, as well as strong coexpression links. Signaling molecules like *TGFB1 *are hypothesized to lie upstream of 'omics measurements, and, thus, the petal at the heart of Figure 4 represents a potential set of intermediaries by which the signal arising from a mutation in *Apc *blossoms into proteome-level manifestations (i.e. the 2D-DIGE targets).

## Conclusions

To understand how a mutation affects information flow in a tumor, one must consider both the proximal and distal signaling effects. Proximally, a mutation in a gene may result in a truncated protein product that affects physical interactions, or it may result in a hyperphosphorylated and active state. These small, upstream effects are then amplified and result in distal changes in signaling, affecting mRNA and protein levels of tens to hundreds of seemingly unrelated nodes. While the field of cell signaling is adept at dissecting the proximal effects of a mutation - mechanistically mapping out perturbed pathways - it has not yet developed the tools to fully understand the distal effects and, more importantly, their connection with more proximal signaling. Indeed, currently available commercial software for network analysis can only associate these distal effects amongst themselves, with no regard to the upstream causative mutation. In this study, we present a method by which the distal effects measured in two 'omics experiments - microarray and proteomics - can be simultaneously leveraged to test network-based hypotheses. After testing the hypotheses (petals) against proteomic evidence, the refined petal subnetworks we present not only reveal the relationship between upstream genetic interference and its downstream effects at the proteomics level, but also allow us to prioritize other cancer-driver genes that are likely to act cooperatively with *Apc *to drive tumorigenesis. This new approach - linking in *silico *predictions with experimental measurements - provides a way forward in mining context-specific pathways that may prove to be useful in identifying pathways active in individual cancer patients.

## Methods

### The Blossom Algorithm

The *Apc *blossom is built using the Blossom algorithm, based on the PathFinder architecture [11]. A recent study compared various frameworks developed for detecting signaling networks [19], and the PathFinder architecture had the best recall rate compared to other available methods, whereas all methods described had a similar precision rate.

In the Blossom algorithm, networks (e.g. pathways) connecting proteins of interest are built by integrating and mining multiple datasets. First, the network of publicly available interactions [20,21] (over 80K interactions) is filtered to remove less reliable interactions, i.e. likely false positives, and, then, new interactions are added to enrich the network to account for missing interactions, i.e. false negatives. To remove false postives, a logistic regression model that incorporates (*i*) the number of times a PPI is observed, (*ii*) coexpression measurements for the corresponding genes, (*iii*) the protein's small world clustering coefficient, and (*iv*) the protein subcellular localization data of interacting partners [22].

Coexpression values (Pearson's correlation coefficient) are calculated from mRNA expression profiles of the laser-capture microdissected epithelium from the *Apc*^*Min*/+ ^mouse (series GSE422 [23]), providing coregulatory information specific to our tissue and organism of interest. The logistic regression model that predicts the validity of interactions is trained on positive (1000 PPIs from the MIPS database [24]) and negative training data sets (1000 randomly selected PPIs not in MIPS, assuming that most interactions are unreliable or irrelevant [11,25]). Repeating these trials 100 times, an optimized cut off point for the probability of a true interaction is set, and a network of reliable interactions is formed (~ 30*K *PPIs).

Finally, false negative interactions are inferred using sequence homology relationships, as it has been shown that similar sequences share similar interaction partners in the same organism [26-29]. An interaction edge is inferred among two proteins if no record of interaction exists, and there exists at least one interaction between the protein families of these two proteins (since sequences sharing similar domains share similar interaction partners [30,31]) (Pfam release 23.0 used [32]).

These steps resulted in a filtered network with predicted edges within which we searched for pathways linking *Apc *and CAN-genes. GO biological process annotations [10] are used to generate functional association rules from know pathways [24,33-35] as outlined in [11]. Association rules are tuples representing a noteworthy relationship, in this case functional relationships, between two interacting proteins. For each protein, leaf terms on the GO term graph are used. In addition, the average absolute coexpression is calculated for each path, and paths are then filtered according to a set threshold (*γ *= 0.6). These rules and parameters are used to evaluate candidate paths for possible occurrences of these rules. The *p*-value, *p_ϕ_*, for a path, *ϕ*, is calculated with the null hypothesis being that every simple path connecting two proteins has a number of association rules associated with these interactions, but the average number of rules on these paths are uniform across various paths. Significant paths, i.e. *p_ϕ _*<*p_threshold_*, are merged into a subnetwork, thus representing a petal in the blossom. An empty set is returned when there is not a significant path.

Formally, let *G*(*V*, *E*) denote the PPI network gathered from publicly available interactions. Also, let *G' *and *G'' *be networks built on the same set of nodes, *V*, using the procedures described above, where false positive interactions, *F*, are removed, *E' *= (*E *- *F*), to obtain *G'*(*V*, *E'*), and a set of additional interactions, *H*, are imputed (based on sequence information) to obtain *E'' *= *E' *∪ *H *forming *G''*(*V*, *E''*).

The objective of the proposed Blossom framework is to find a petal for a given protein *c_a _*∈ *V *(in our case, *Apc*) and each protein *c_i _*in the candidate set of proteins *C *⊂ *V *(CAN-genes). To reduce the search space, Blossom employs a network diameter heuristic. Namely, for each node pair (*c_a _*and *c_i_*), let *d_i _*denote the shortest path between *c_a _*and *c_i _*in *G*(*V*, *E*) (PPI network without inferred edges). For each *c_i _*∈ *C*, we then search *G'*(*V*, *E'*) for every path that connects *c_a _*to *c_i _*with path length smaller than *d_i _*that connect *c_a _*and *c_i_*. This guarantees at least one path for consideration if the two nodes are connected.

The paths on the network are discovered using all paths depth first search (*AllPathsDFS*), where every path connecting *c_a _*and *c_i _*that is less than *d_i_*, Φ*_i _*, is identified. In the final step of the algorithm, these paths are compared against the null distribution for significance. For the shortest path calculation, a single-source shortest path solution is used (e.g. Dijkstra's algorithm). The Blossom algorithm's run time is the same as the all-paths depth first search: $\text{O(}V^{d_{max}}\text{)}$ where $d_{max}=\max_{c_{i}\in C}d_{i}$.

**Input**: *c_a_*, *C*, *G*(*V*, *E*), *G''*(*V*, *E''*), *p_threshold_*, *γ*

**Output**: $G_{c_{a}}$

**foreach ***c_i _*∈ *C *do

   *d_i _*= ShortestPathDistance(*G*(*V*, *E*), *c_a_*, *c_i_*);

   **if ***d_i _*= = ∞ **then**

      *d_i _*= ShortestPathDistance(*G''*(*V*, *E'*), *c_a_*, *c_i_*);

   **end**

   Φ*_i _*= AllPathsDFS(*G''*(*V*, *E''*), *c_i _*, *c_a_*, *d_i_*);

   **forall the ***ϕ *∈ Φ*_i _***do**

      **if ***r*(*ϕ*) ≤ *γ and p_ϕ _*<*p_threshold _***then**

         $G_{c_{a}}=G_{c_{a}}\cup \phi$.

      **end**

   **end**

end

**Algorithm 1**: The Blossom algorithm that returns the blossom network for protein *c_a_*.

### Plucking Petals: Testing Bimodality of Coexpression

For a particular petal, a single node perturbation (e.g. a mutation at *Apc*) within the petal itself will perturb pathways that are expected to associate with the given petal more strongly than others, assuming that the network predictions were accurate. To identify the best petal in the *Apc *blossom, we employed a mouse mutant, *Apc*^*1638N*+/-^, representing a perturbation at the stamen. The transcript and protein levels of *Apc *itself have been verified in previous studies [18]; in this study, we were interested in distilling the myriad downstream effects into a coherent set of candidate pathways. As proteins are the ultimate mediators of function, targets from proteomic experiments - such as label-free, or, in our case, 2 D DIGE - represent an ideal dataset for assessing the downstream effects of such perturbations. However, proteomic technologies often sample the most abundant quartile of proteins [36], while cancer network predictions - such as those in the *Apc *blossom - often focus on low-abundance signaling proteins.

In order to make inferences about identified petals, a relational map must be used to connect the proteomic targets to the petal of interest. Coexpression networks are currently the most informative and accessible mapping available, as proteins correlated at the mRNA-level are hypothesized to be coregulated.

Thus, for a hypothesized petal, *P*, mRNA coexpression (Pearson's correlation coefficient) was calculated between the nodes, *i *∈ *P *, and the 2D-DIGE targets, *d *∈ *D *(where *D *⊂ *S *and *S *is the set of all genes on the array) measured in the *Apc*^*1638N*+/- ^mouse intestinal epithelium. The 2D-DIGE targets' Mascot DAT files are available through the Proteomics Identifications Database (accession number 10638) [37].

*Apc*^*1638N*+/- ^microarray data is available through the Gene Expression Omnibus (GSE19338) [38]. Two fractions, representing crypts and villi, were available with four samples in each group (eight samples each, wild-type and *Apc*^*1638N*+/-^). Though the mild phenotype of the *Apc*^*1638N*+/- ^mouse appears to result in a low signal - in stark contrast to that observed from *Apc*^*Min*/+ ^mice - many molecular changes are still measurable, as evidenced by the 'omic experiments. The proteins identified within each fraction were pooled to arrive at a set of 31 2D-DIGE targets shown on the periphery of Figure 4 (see [17] for detailed methods). Robust Multiarray Averaging was used to normalize mRNA expression measurements, and differential expression was calculated between the eight mutant samples versus the eight wild-type samples. For coexpression, the wild-type and *Apc*^*1638N*+/- ^microarray data were normalized by dChip [39] to avoid artificially inflating coexpression values [40].

Additionally, mRNA coexpression is more informative for nodes that are known to be differentially expressed, as these nodes are regulated differently between wild-type (*WT*) and mutant tissue (*MT*); a node with low differential expression may have many coexpression linkages simply due to its uniform expression profile over the samples, which is shared by the majority of genes (as most genes are not differentially regulated). To focus on genes with strong levels of both coexpression and differential expression, we compute the *active coexpression *as follows:

$$\vec{r_{i}}\text{'}=\alpha_{i}\cdot \vec{r_{i}}$$

Where $\vec{r_{i}}$ is the vector of coexpression between node *i *(in petal *P*) and all other genes on the array; α*_i _*is the *activity *of node *i*, defined as the scaled, absolute differential expression:

$$\begin{array}{c} t_{i}=\frac{\mu_{MT,i}-\mu_{WT,i}}{\sqrt{\frac{\sigma_{MT,i}^{2}}{n_{MT,i}}+\frac{\sigma_{WT,i}^{2}}{n_{WT,i}}}}\\ \alpha_{i}=\frac{\left|t_{i}\right|}{\arg\max t_{i}} \end{array}$$

**Figure 5:**
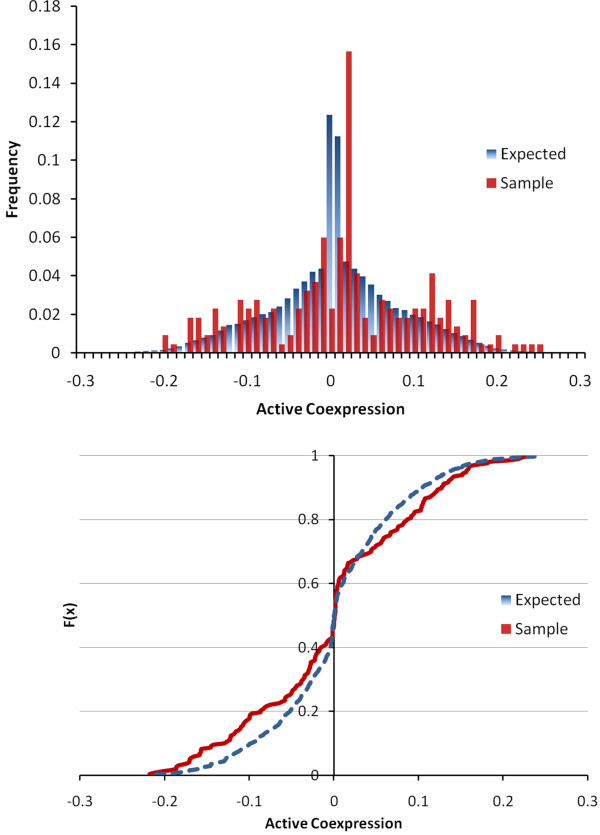
**Bimodality of Coexpression**. **(A) **Active coexpression, $\vec{r_{i}}$ values for the expected and sample distributions for an example petal. Note the extreme peakedness around the origin. **(B) **The cumulative distribution functions (CDFs) of the expected and sample values are shown. This particular petal has more negative and positive active correlations to the 2D-DIGE targets than to the rest of the genes on the array. To assess the bimodality, *β*, of the data, we examine the area between the two CDFs, as well as the center of mass of this area.

Where *μ*_*MT*,*i *_is the average expression of a gene, *i*, across the samples in the mutant, *MT *(in our case, *Apc*^*1638N*+/-^), and *σ*^2 ^is the associated variance; these parameters are defined respectively for the wild-type (*WT*) samples. The active coexpression matrix, *R'*(*P*, *D*), between a given petal, *P*, and the 2D-DIGE targets, *D*, is then vectorized, *vec*(*R'*(*P*, *D*)). The distribution of *vec*(*R'*(*P*, *D*)) is expected to be leptokurtic (i.e. higher peak, fatter tails), as it is a product of a normal and a folded normal distribution (see Figure 5A). With coexpression measurements, we are particularly interested in the tails of the distribution, as these are expected to exhibit two modes - one positive and one negative - if subgroups of coexpressed 2D-DIGE targets exist. Thus, we developed a measure of bimodality, *β*:

$$\begin{array}{c} \Delta F_{P}(x)=F_{P,D}(x)-F_{P,S}(x)\\ \beta_{P}=l_{x<0}\int_{-\infty }^{0}\Delta F_{P}(x)dx+l_{x\geq 0}\int_{0}^{\infty }\Delta F_{P}(x)dx \end{array}$$

*F_P,D _*is the empirical cumulative distribution function (CDF) for *vec*(*R'*(*P*, *D*)) over the range of active coexpression values, *x*; *F_P,S _*is the empirical CDF for *vec*(*R'*(*P*, *S*)) i.e. the expected active coexpression to all genes on the array; and the sample deviation, Δ*F_P _*, is simply the difference of the two CDFs. *l*_*x*<0 _is the moment arm of the distribution defined classically as:

$$l_{x<0}=\frac{\sum_{i,x_{i}<0}\Delta F_{P}(x_{i})\cdot x_{i}}{\sum_{i,x_{i}<0}\Delta F_{P}(x_{i})}=\frac{\int_{-\infty }^{0}\Delta F_{P}(x)\cdot x\;dx}{\int_{-\infty }^{0}\Delta F_{P}(x)dx}$$

And *l*_*x*≥0 _is defined similarly. Thus, *l*_*x *< 0 _and *l*_*x*≥0 _represent the centers of mass for the negative and positive active coexpression values' deviation from the expected distribution (Figure 5B). The bimodality, *β_P_*, then, is simply the torque of the distribution, Δ*F_P_*(*x*), around the center: negative values of *β_P _*indicate a clockwise skewing of the tails, with greater mass distributed at extreme (high and low) values of *r' *than the background; positive values of *β_P _*indicate a counterclockwise skew, where the sample distribution is more leptokurtic than the background, and, hence, possesses less correlation than expected. Further insight can be gained by noting that the denominator of the center of mass, *l_x_*, cancels out, leaving:

$$\begin{array}{c} \beta_{P}=\int_{-\infty }^{0}\Delta F_{P}(x)\;x\;dx+\int_{0}^{\infty }\Delta F_{P}(x)\;x\;dx\\ =\int_{-\infty }^{\infty }x(F_{P,D}(x)-F_{P,S}(x))\;dx\\ =\int_{-\infty }^{\infty }x\int_{-\infty }^{x}f_{P,D}(y)-f_{P,S}(y)\;dy\;dx \end{array}$$

Changing the order of integration allows us to formulate *β_P _*in terms of the probability density functions (PDFs) of our targets, *f_P,D_*(*x*), and the background, *f_P,S_*(*x*):

$$\begin{array}{c} \beta_{P}=-\frac{1}{2}\int_{-\infty }^{\infty }x^{2}(f_{P,D}(x)-f_{P,S}(x))dx\\ =-\frac{1}{2}(E(x_{P,D}^{2})-E(x_{P,S}^{2})) \end{array}$$

Where *E*(·) indicates the expectation. Thus, we see that *β_P _*is the difference between the second moments of the two distributions (or the difference of their variances, if both distributions are centered at zero).

While this ultimate formulation of *β_P _*is statistically simple, we present the initial formulation - in terms of the center of mass and torque - to provide an intuitive understanding of its motivation and meaning. As mentioned, we use the empirical CDF/PDF to calculate *β_P_*. We calculated the significance, *p*, of *β_P _*for a network-petal, *P*, as follows:

$$p=\frac{\#\beta_{rand}<\beta_{P}}{\#\beta_{rand}}$$

With *β_rand _*being the bimodality for a randomly selected set of candidate 2D-DIGE targets; 10000 such sets (of cardinality equal to that of *P*) were generated. Then, the null hypothesis is that the coexpression pattern between the network-petal and the proteomic targets is random, and the p-value is the probability of attaining at least a value of |*β_P_*| via stochastic generation of 2D-DIGE targets.

## Authors' contributions

GB and VP designed, carried out the experiments and drafted the manuscript. GB and VP equally contributed to this article. MRC supervised the study. All authors read and approved the final manuscript.

## Supplementary Material

Additional file 1 **Additional table listing petals identified**. The petal subnetworks identified and the bimodality scores calculated against the proteomics targets for each petal are listed in this file. Click here for file
