# Supplementary material for: PETALS: Proteomic Evaluation and Topological Analysis of a mutated Locus' Signaling
Source: BMC Bioinformatics. 2010 Dec 13;11:596. doi: 10.1186/1471-2105-11-596 (PMC3016410; doi:10.1186/1471-2105-11-596)
Supplement: Additional file 1 — Additional table listing petals identified. The petal subnetworks identified and the bimodality scores calculated against the proteomics targets for each petal are listed in this file. [file 1471-2105-11-596-S1.PDF]

Additional File 1

Table S1: The petal subnetworks identified and the bimodality scores calculated against the proteomics targets for each petal are listed

Rank	Petal	Bimodality (β)	p-value	Petal Size	Petal Nodes
1	*Hapln1*	-0.00037	0.0068	12	*Acvrl1, Apc, Ctnnb1, Egfr, Fgfr1, Hapln1, Mmp2, Mmp3, Mmp9, Src, Tgfb1, Tgfbr1*
2	*Kras*	-0.001257	0.0157	7	*Apc, Bcl2, Bcl2l10, Cdk2, Kras, Mapk8, Prkaca*
3	*Prkd1*	-0.000239	0.0167	35	*Apc, Apc2, Ar, Bcl2l1, Casp8, Ctnnb1, Cxcr4, Egfr, Fyn, Gna13, Grik2, Igf1, Igf1r, Kdr, Lyn, Map4k1, Mapk8, Nr5a1, Pdgfrb, Pecam1, Pik3r1, Pkd1, Prkaca, Prkd1, Psen2, Ptk2, Ptpn6, Rxra, Shc1, Smad3, Src, Stat1, Tbl1x, Tcf3, Tgfbr1*
4	*Mmp2*	-0.000263	0.0198	11	*Acvrl1, Apc, Csnk2a1, Ctnnb1, Fgfr1, Itgav, Mmp2, Prkaca, Ptk2, Src, Tgfb1*
5	*Exoc4*	-0.000232	0.0202	8	*Apc, Cdh2, Ctnnb1, Exoc4, Fyn, Gna13, Grin2b, Src*
6	*P2rx7*	-0.000328	0.0212	11	*Acta1, Actb, Apc, Ar, Ctnnb1, Egfr, P2rx7, Ptk2, Rac1, Smad3, Svil*
7	*Evl*	-0.000125	0.0223	6	*Apc, Casp8, Ctnnb1, Evl, Fyn, Src*
8	*Sfrs6*	-0.000209	0.0245	16	*Apc, Clk1, Ctnna1, Ctnnb1, Egfr, Gna13, Pik3r1, Prkaca, Psen2, Ptk2, Ptk2b, Rxra, Sfrs6, Smad4, Src, Srpk1*
9	*Epha3*	-0.000197	0.0264	8	*Apc, Ctnnb1, Epha3, Nr5a1, Pdgfrb, Ptk2, Shc1, Src*
10	*Lmo7*	-0.000077	0.0287	3	*Apc, Ctnnb1, Lmo7*
11	*Syne1*	-0.000267	0.0294	10	*Actc1, Apc, Cdk5r1, Ctnnb1, Egfr, Gna13, Rxra, Smad3, Src, Syne1*
12	*Hist1h1b*	-0.00015	0.0382	35	*Acvrl1, Apc, Ar, Axin1, Axin2, Bcl2, Bcl2l1, Cdh2, Cdk2, Chuk, Ctnnb1, Cxcr4, Egfr, Fgfr1, Fyn, Gna13, Hist1h1b, Kdr, Lox, Mapk8, Melk, Met, Pdgfrb, Pik3r1, Pkd1, Prkaca, Psen2, Ptk2, Rxra, Shc1, Smad2, Smad3, Src, Stat1, Tgfbr1*
13	*Slc29a1*	-0.000132	0.0437	6	*Apc, Cebpa, Ctnnb1, Pax3, Sox10, Slc29a1*
14	*Nf1*	-0.000088	0.0566	6	*Apc, Ctnnb1, Grin1, Grin2d, Nf1, Tbl1x*
15	*Pkhd1*	-0.000078	0.0584	10	*Apc, Ccr5, Ctnnb1, Cxcr4, Ednrb, Gna13, Jak2, Pkhd1, Ptk2, Stat1*
16	*Lrp2*	-0.000203	0.0672	14	*Apc, Cdh3, Csnk2a1, Ctnnb1, Fyn, Kdr, Lrp2, Ncoa2, Pik3r1, Ptk2, Rxra, Src, Tbl1x, Tgfbr1*
17	*Smad3*	-0.000413	0.0908	3	*Apc, Ctnnb1, Smad3*
18	*Smad4*	-0.000021	0.1120	3	*Apc, Ctnnb1, Smad4*
19	*Pknox1*	-0.000185	0.1142	11	*Apc, Axin2, Csnk2a1, Ctnnb1, Dvl3, Kdr, Pknox1, Psen2, Smad3, Src, Tgfbr1*
20	*Cd46*	-0.000048	0.1606	13	*Apc, Cd46, Cdh2, Ctnnb1, Cxcr4, Egfr, Gna13, Nr5a1, Psen2, Ptk2, Shc1, Src, Tgfbr1*
21	*Smad2*	0.000011	0.6705	3	*Apc, Ctnnb1, Smad2*
22	*Runx1t1*	0.000026	0.7353	4	*Apc, Ctnnb1, Runx1t1, Tcf3*
23	*Tcf7l2*	0.000074	0.9546	3	*Apc, Ctnnb1, Tcf7l2*
24	*Ephb6*	0.000755	0.9973	5	*Apc, Ctnnb1, Egfr, Ephb6, Ptk2*
